# Sources of Differences in Right-Wing Authoritarianism and Social Dominance Orientation from Adolescence to Adulthood: A Multi-Cohort Twin Family Study

**DOI:** 10.1007/s10519-025-10242-0

**Published:** 2025-11-03

**Authors:** Christian Kandler, Jana Instinske, Edward Bell

**Affiliations:** 1 Department of Psychology, Bielefeld University, Bielefeld, Germany; 2 Department of Sociology, University of Western Ontario, London, ON Canada

**Keywords:** Right-Wing authoritarianism, Social dominance orientation, Multi-cohort nuclear twin family model, Genotype-environment correlation

## Abstract

Past research indicates that genetic and environmental sources contribute to Right-Wing Authoritarianism (RWA) and Social Dominance Orientation (SDO). However, less is known about the differences between the sources of variance in RWA and SDO, and how these sources differ across different developmental stages. Based on data from 1440 twin families, including 1198 complete twin pairs, 435 non-twin full siblings, and 2016 parents of twins from the German TwinLife project, nuclear twin family modeling was used to estimate different genetic and environmental variance components of RWA and SDO, and to examine the extent to which the variance components vary across three age cohorts of twins (average age 15, 21, and 27 years). We hypothesized increasing levels of genetic variance across age cohorts, reflecting more active genotype-environment transactions with development, and declining levels of passive genotype-environment correlation (*r*GE). The model analyses yielded additive and nonadditive genetic factors as well as influences shared in families due to non-genetic intergenerational transmission from parents to offspring and significant passive *r*GE for RWA. For SDO, passive *r*GE components, nonadditive genetic components and non-genetic intergenerational transmission were negligible. However, significant individual environmental sources and those only shared by twins were found for both RWA and SDO. Inconsistent with our expectations, passive *r*GE and genetic variance did not vary significantly across age cohorts. Counterintuitively, the influence of twin-specific shared environmental factors on RWA was larger in adulthood than adolescence, suggesting increasingly relevant environmental influences across developmental periods. These results have important implications for socio-developmental theories on socio-political attitudes.

## Introduction

Right-Wing Authoritarianism (RWA) and Social Dominance Orientation (SDO) have been proposed as two personality-related constructs that describe fundamental individual differences in socio-political attitudes. RWA is thought to encompass an individual’s preference for collective security while maintaining established social conventions and rejecting social change (Altemeyer 1981), whereas SDO captures a preference for intergroup stratification while favoring one’s own group over other groups (Pratto et al. 1994). The two constructs show some theoretical and empirical overlap, but also provide unique information on socio-political attitudes (Duckitt and Sibley 2010; Sibley and Duckitt 2008). Since individual differences in RWA and SDO predict a variety of socially significant outcomes, such as support for authoritarian politics, prejudice against minorities, and endorsement of discrimination (e.g., Duckitt and Sibley 2010; Kandler et al. 2015), these two constructs are important within the social sciences. Understanding why individuals differ in their levels of RWA and SDO can help us make sense of political polarization and the rise of extremist movements, and may also provide a foundation for efforts to reduce political tensions and curb discrimination.

Although previous behavioral genetic research generally indicates that RWA and SDO are influenced by both genetic and environmental factors (de Vries et al. 2022; Hatemi et al. 2014; Kleppestø et al. 2024), it is mostly based on classical twin studies. Therefore, prior studies rarely distinguished between different genetic and environmental sources nor did they examine how these sources covary. Questions remain as to the specific nature of those factors and how they may combine and relate to each other. A related question that remains unanswered pertains to whether those influences vary throughout the life course, especially during the formative years of socio-political development. If the relevance of genetic and environmental factors on the variance in RWA and SDO shifts across age groups, influences that are effective in adolescence may be ineffective later on. In the present study, we critically addressed the empirical questions about the role of genetic and environmental influences. In doing so, we expanded on previous research (1) by using a nuclear twin family design that included data from twins, their non-twin siblings, and their parents, and (2) by analyzing data from three different age cohorts of twins that spanned the period from adolescence to young adulthood—a time that is deemed to be a crucial stage in life for the formation of socio-political orientations (Jennings et al. 2009). This allowed us to disentangle different genetic sources (e.g., additive and nonadditive factors) and environmental sources (e.g., intergenerational transmission and sibling-specific effects), while accounting for assortative mating and genotype-environment correlation. It also enabled us to test different theories of genotype-environment interplay during key stages of socio-political development.

### Different Sources for RWA and SDO?

Several studies have consistently reported moderate to substantial genetic influences on RWA, accounting for 30% to 50% of its variance, while environmental influences shared by twins have explained 10% to 30% (e.g., Funk et al. 2013; Kleppestø et al. 2024; Lewis and Bates 2014; Ludeke and Krueger 2013; McCourt et al. 1999; Nacke and Riemann 2023). Somewhat lower genetic and shared environmental contributions (on average about 30% and 10% of the variance, respectively) have been found for SDO (de Vries et al. 2022; Kleppestø et al. 2024; Kandler et al. 2015; Kandler 2015; Nacke and Riemann 2023). Hence, individual-specific environmental factors (including error of measurement) appear to account for most of the differences in SDO, suggesting that extra-familial sources not shared by family members may be more important for SDO than for RWA.

After controlling for error of measurement, familial resemblance based on reliable latent variable scores across self- and informant reports was found to be considerable for both RWA and SDO (Kandler et al. 2016). In particular, the within-generational similarity of monozygotic (MZ) and dizygotic (DZ) twins, corrected for random error of measurement and systematic rater specificity, indicated substantial environmental influences shared by twins on variance in both RWA (*r*_MZ_ = 0.88 and *r*_DZ_ = 0.60) and SDO (*r*_MZ_ = 0.66 and *r*_DZ_ = 0.62). However, the correlations between twins’ parents were also substantial, being *r* = .45 for RWA and *r* = .42 for SDO. This is indicative of *assortative mating*, which occurs if parents have not mated in an entirely random fashion, but instead on the basis of their phenotypic resemblance to one another (e.g., Briley et al. 2019). A recent meta-analysis by Horwitz et al. (2023) yielded considerable partner correlations for political values (*r*_meta_ = 0.58), supporting the idea that assortative mating could play a crucial role for RWA and SDO.

Assortative mating increases the genetic resemblance of twins’ parents and thus the genetic resemblance of their DZ twin offspring, but not the genetic resemblance of their already genetically identical MZ twin offspring. If assortative mating is of relevance but is not accounted for in variance decomposition analyses, shared environmental influences will be overestimated and additive genetic influences underestimated. Nuclear twin family models (NTFMs) based on data from twins and their parents allow one to examine the resemblance of twins’ parents on the trait of interest, and thus to account for assortative mating effects. The NTFM analyses of Kandler and colleagues (2016) showed that, after taking assortative mating into account, the estimates of twins’ shared environmental influences on variance in RWA and SDO were reduced and primarily generation-specific, indicating the importance of joint socialization outside the parental sphere, such as shared peer-group exposure. Since no data from non-twin siblings were available in this previous study, the authors were unable to distinguish between sibling-specific and twin-specific shared influences. A substantial twin-specific effect would signal that being a twin per se may create a distinct socialization context that shapes socio-political attitudes.

Given their moderate correlation (Sibley and Duckitt 2008), RWA and SDO might show some similar properties regarding their sources of variance. Previous studies suggest substantial genetic but little to no environmental overlap, indicating that environmental effects are rather construct-specific (e.g., Lewis and Bates 2014; Nacke and Riemann 2023). Kandler et al. (2016) found that controlling for their shared variance with RWA, individual differences specific to SDO were not significantly influenced by genetic factors, but primarily attributable to unique environmental factors not shared within families. For RWA, by contrast, genetic factors were found to form a considerable source of individual differences. However, other studies have concluded that the level of RWA might be more malleable by societal circumstances than is the case with SDO (Zubielevitch et al. 2023). Given this apparent inconsistency in the literature, we wanted to find out whether certain genetic and environmental factors are of different importance for RWA and SDO.

Not only different genetic and environmental sources but also different kinds of nonrandom links between genetic and environmental factors could be of relevance to the formation of individual differences in RWA and SDO. Such nonrandom links include, for instance, passive genotype-environment correlations (*r*GE). *Passive r*GE refers to situations in which children, who share substantial portions of their genotype with their parents, are exposed to rearing environments provided by their parents that are linked to their parents’ genetically influenced traits and behaviors. This passive rGE can be estimated with NTFM analyses. In the case of RWA and SDO, parents may provide genetically influenced environmental cues that reinforce their children’s natural inclinations toward those two characteristics. One study that used data from twins and their parents in a NTFM found evidence of passive *r*GE for RWA but not for SDO, even though the sample included a broad age-range of adult participants (Kandler et al. 2016).

### Different Sources for Different Ages?

Developmental theories suggest a declining impact of within-family influences including passive *r*GE on individual differences during psycho-social development, likely due to increasing self-determination (Scarr 1992, 1993; Scarr and McCartney 1983). Therefore, in the late teen years, diminishing effects of environments shared by twins and lower passive *r*GE can be expected, especially after children have grown up and left the familial household, while other types of *r*GE become more important. For example, politically conservative young adults may choose news sources or friendship circles and elicit social responses such that their genetically influenced conservative leanings are welcomed and affirmed. These types of *active* or *reactive r*GE (the latter is also referred to as *evocative r*GE) occur in situations in which individuals’ genetically-based predispositions lead them to choose environments that provide a good fit for those predispositions, or lead them to provoke or construe their environmental circumstances in a particular way (Plomin et al. 1977; Kandler et al. 2021, 2024). They are thought to become increasingly relevant during adolescence and early adulthood, as individuals in this period of life begin to physically and psychologically distance themselves from the parental home environment and transact with their broader environments more autonomously (Kandler et al. 2019).

One study that examined correlates of both RWA and SDO, such as general left-right political orientations, across two age-groups found a decrease in the extent of passive *r*GE from adolescence to young adulthood (Hufer et al. 2020). Hufer et al. also reported a greater additive genetic variance component for political orientation for young adults than for adolescents, a finding consistent with the idea that genetically driven extrafamilial socialization increases in young adulthood through greater active and evocative *r*GE (Scarr and McCartney 1983; Kandler et al. 2021). This increase in additive genetic effects from adolescence to young adulthood is also consistent with previous findings on socio-political attitudes. For overall liberalism-conservatism, Hatemi et al. (2009) reported minimal additive genetic variance in adolescents but substantial levels of it after age 21. A similar pattern was observed by Eaves et al. (1997) for political conservatism.

As RWA and SDO show conceptual parallels and empirical links to general left-right political orientations and conservatism, comparable age trends can be expected for both. A recent cohort-sequential study on the developmental trends in RWA and SDO found that, on average, the levels of both RWA and SDO tended to increase with age, although cohort differences and, thus, contextual influences seemed to play a greater role for RWA in this respect (Zubielevitch et al. 2023). However, research from a developmental perspective on the extent to which genetic and environmental variance and covariance account for individual differences in RWA and SDO has, to our knowledge, never been conducted. The present extended twin family study with twins at three different developmental stages (adolescence, emerging adulthood, and young adulthood) endeavored to fill that gap.

### The Current Study

The key aims of this research were to disentangle different genetic and environmental sources of variance in RWA and SDO, and to determine whether the importance of those sources differed between the two constructs and across three age cohorts. The youngest of these three age cohorts consisted of twins born in the years 2003–2004 (*M*_*age*_ ≈ 15), which we designated as “adolescents.” The intermediate cohort, with twins born in 1997–1998 (*M*_*age*_ ≈ 21), was classified as “emerging adults.” The oldest cohort, with twins born from 1990 to 1993 (*M*_*age*_ ≈ 27), was described as “young adults.” Non-twin siblings who were roughly the same age were also investigated. Crucially, the study also incorporated data from twins’ parents, who are at a different developmental stage than their offspring. This broader family design is essential when investigating different sources of siblings’ similarities and differences in the offspring generation. These sources include (1) within-generational sibling- and twin-specific similarities due to nonadditive genetic and age- or generation-specific environmental influences, (2) the role of intergenerational genetic and environmental transmission, (3) nonrandom links between genetic factors and family environment (i.e., passive *r*GE), while (4) taking nonrandom spouse similarity (i.e., assortative mating) of twins’ parents into account (Hufer et al. 2020; Keller et al. 2009a, b).

Unlike classical twin models, NTFMs can consider the simultaneous relevance of nonadditive genetic factors and environmental influences shared by twins, which otherwise would result in biased estimates of genetic and environmental components (Instinske and Kandler 2024; Keller et al. 2009b). With the use of a more fine-grained NTFM analysis, we looked for potential differences in the sources of variance in RWA and SDO, while taking into account (1) construct-specific variance in RWA and SDO with the use orthogonal factor scores, and (2) variance adjusted for measurement error with the use of latent variable scores. To this end, we compared the results from the analyses using correlated mean scores, orthogonal factors scores, and latent variable scores to ensure the robustness of the findings across different measures.

Fitting multi-cohort nuclear twin family models (MC-NTFMs) based on three different age cohorts further allowed us to compare the genetic and environmental contributions to the variance from adolescence to young adulthood. Based on the findings from previous research discussed above, we hypothesized that the role of passive *r*GE decreases with higher age across the three age groups for RWA (Hypothesis 1a) and SDO (Hypothesis 1b). In addition, we hypothesized that at the same time the variance attributable to genetic sources increases with age across the three age cohorts for RWA (Hypothesis 2a) and SDO (Hypothesis 2b), which would be indicative of greater effects originating from active and evocative *r*GE. Both hypotheses were preregistered as part of an overarching project (https://osf.io/6asfj/).

## Methods

### Study Design and Data Collection Procedures

We analyzed data from the German twin family panel TwinLife, a genetically informative, longitudinal study that tracks individual development and social inequality through the life course (Diewald et al. 2024; Hahn et al. 2016). MZ and same-sex DZ twin pairs from four different birth cohorts as well as their parents and, if available and old enough, non-twin siblings were interviewed. Participants were randomly selected based on national population registration offices, which produced a representative sample of German twin families. The TwinLife core project encompasses five waves of data collection that began in 2014 and concluded in 2024 (see also www.twin-life.de/documentation/).

Participants have been surveyed on various constructs, alternating between face-to-face interviews in the household and computer-assisted telephone interviews. At the time of the third face-to-face interview, conducted in the period from 2018 to 2020, information on RWA and SDO was assessed for participants 13 years of age and older. The constructs were not measured again in any other data wave. See Rohm et al. (2023) for details on the study design of TwinLife.

To determine if a twin pair was monozygotic or dizygotic, a standardized zygosity questionnaire was used at the time of the twins’ first participation. The questionnaire comprised items concerning the respondent’s perception of the twin’s physical similarity to the respective co-twin. For the two older age-cohorts, the twins’ themselves answered the relevant questions, whereas for the younger age-cohort, the questionnaire was completed by the twins’ parents. Compared to DNA-based classifications, the standardized questionnaire provides high rates of correct zygosity classification, ranging from 92% to 97% (Lenau et al. 2017). The present study assessed zygosity using the questionnaire data, and those classifications were checked (and corrected where necessary) using DNA information in cases for which such information was available.

### Sample

Overall, data from 1440 twin families were available, which involved 5089 different participants who provided self-reports on RWA and SDO. We aimed to use the highest sample size possible based on the data available. Therefore, we included data from twin pairs for which information on RWA or SDO was available for at least one of the co-twins. The resulting sample includes 1270 MZ twins (58% female) from 687 pairs, with 583 pairs for which data from both twins were available, and 1368 DZ twins (53% female) from 753 pairs, with 615 complete pairs. From these twins, 1129 were designated as adolescents (Age: *M* = 15.04, range: 14–16), 820 as emerging adults (Age: *M* = 21.09, range: 20–22), and 689 as young adults (Age: *M* = 27.11, range: 25–29).

**Table 1:** Descriptive statistics of the twin family sample

	Birth cohort and zygosity						
	2003–2004		1997–1998		1990–1993		
	MZ	DZ	MZ	DZ	MZ	DZ	Total
*N* of twin families	248	328	219	237	220	188	1440
*N* of twin a	242	323	204	219	199	160	1347
*N* of male twin a	108	166	83	91	77	67	592
*N* of female twin a	134	157	121	128	122	93	755
*N* of twin b	244	320	189	208	192	138	1291
*N* of male twin b	110	166	76	87	76	66	581
*N* of female twin b	134	154	113	121	116	72	710
*N* of complete twin pairs	238	315	174	190	171	110	1198
*N* of non-twin sibs	88	109	76	74	47	41	435
*N* of male non-twin sibs	40	63	42	38	15	24	222
*N* of female non-twin sibs	48	46	34	36	32	17	213
*N* of mothers	235	315	165	190	150	118	1173
*N* of fathers	168	248	121	145	90	71	843
Age mean of twins	15.04	15.04	21.12	21.05	27.17	26.99	20.08
Age range of twins	14–16	14–16	20–22	20–22	25–29	25–29	14–29
Age mean of non-twin sibs	18.53	18.39	22.39	22.80	27.30	27.32	21.67
Age range of non-twin sibs	13–28	13–27	13–32	13–38	14–38	18–38	13–38
Age mean of mothers	46.78	48.00	52.15	53.15	56.49	57.52	51.22
Age range of mothers	33–58	35–62	43–62	44–67	46–67	46–67	33–67
Age mean of fathers	50.47	51.14	54.91	55.71	59.43	59.46	53.92
Age range of fathers	34–77	38–77	45–68	43–78	47–70	46–77	34–78

*MZ* Monozygotic, *DZ* Dizygotic

In addition to the twins, RWA and SDO self-report data from 435 non-twin full siblings were available, including 197 siblings (48% female) of adolescent twins, 150 siblings (47% female) of emerging adult twins, and 88 siblings (56% female) of young adult twins. From the 1440 twin families, 1173 mothers (81%) and 843 fathers (59%) provided RWA and SDO self-reports. See Table 1 for numbers of twins and their family members by twins’ birth cohort and zygosity as well as sex distributions and age statistics.

### RWA and SDO Measures

RWA and SDO were assessed by means of self-reports. RWA was measured using a four-item version of the German Right-Wing Authoritarianism Scale RWA³D (Funke 2005; Hebler et al. 2001; example item: “The real keys to the ‘good life’ are obedience, discipline, and virtue.”). A German variant (Kämpfe 2002) of the four-item scale for measuring Social Dominance Orientation (Pratto et al. 1994) was used to assess SDO (example item: “If some groups kept to themselves, we would have fewer problems.”). Participants were asked to respond to the items contained in both instruments on a 5-point-Likert scale according to their level of agreement (1: “disagree very strongly” – 5: “agree very strongly”). Two RWA items and one SDO item were negatively formulated regarding the construct and thus had to be reversely coded. For more details on the measurement instruments see the TwinLife data documentation website (https://www.twin-life.de/documentation/).

Internal consistency estimates based on Cronbach’s α (Cronbach 1951) and McDonald’s total ω (McDonald 1999) were α = 0.59 and ω = 0.60 for RWA and α = 0.50 and ω = 0.54 for SDO. Given the brevity of the RWA and SDO measures, and the need for each measure to encompass the theoretical bandwidth of the constructs, the rather low internal consistency can be treated as acceptable. In addition, confirmatory factor analyses (CFA), allowing for two dimensions representing RWA and SDO as well as systematic residual correlations between the three recoded items to consider systematic method variance, were run. To evaluate overall model fit, the comparative fit index (CFI; Bentler 1990) and the root mean square error of approximation (RMSEA; Steiger and Lind 1980) were used, with CFI > 0.95 and RMSEA < 0.05 indicating good fit, and CFI > 0.90 and RMSEA < 0.08 suggesting adequate fit (Hu and Bentler 1999; Steiger 2007). The CFA supported the two-dimensional structure with acceptable model fit to the data (CFI = 0.925, RMSEA = 0.067) and thus provided factorial validity of the RWA and SDO scores.

The RWA and SDO measures were tested for different levels of measurement invariance (MI; Putnick and Bornstein 2016) across the two zygosity groups, the three age cohorts, and five samples representing different groups of family members (i.e., firstborn twins, second-born co-twins, non-twin siblings, mothers, and fathers). To compare the model fit of different MI levels, we used the common descriptive criterion to identify lack of MI: A more constrained model should not show a decrease in the CFI value > 0.01 (Cheung and Rensvold 2002). Table 2 summarizes the MI model tests. In sum, strict MI could be assumed across zygosity groups and metric MI could be assumed across twins’ birth cohorts and the five family members. Since metric MI is the basic prerequisite for ensuring that the same constructs were measured in each subsample with equal variance in each indicator, the basic fundament for variance-covariance decomposition in the NTFM analyses was fulfilled.

**Table 2:** Measurement invariance (MI) testing of RWA and SDO measures

Models	χ²	df	Δχ²	Δdf	*p*	RMSEA	CFI	ΔCFI
Measurement invariance testing across zygosity groups								
Configural MI	427.09	32				0.070	0.920	
Metric MI	442.75	38	15.66	6	0.016	0.065	0.918	0.002
Scalar MI	450.95	44	8.20	6	0.224	0.060	0.918	0.000
**Strict MI**	**458.40**	**52**	**7.45**	**8**	**0.489**	**0.055**	**0.918**	**0.000**
Measurement invariance testing across twins’ birth cohorts								
Configural MI	416.40	48				0.067	0.925	
**Metric MI**	**445.88**	**60**	**29.47**	**12**	**0.003**	**0.062**	**0.921**	**0.004**
Scalar MI	550.65	72	104.77	12	< 0.001	0.063	0.903	0.018
Strict MI	569.47	88	18.82	16	0.278	0.057	0.902	0.001
Measurement invariance testing across family members								
Configural MI	457.88	80				0.068	0.925	
**Metric MI**	**513.55**	**104**	**55.67**	**24**	**< 0.001**	**0.062**	**0.919**	**0.006**
Scalar MI	855.46	128	341.91	24	< 0.001	0.075	0.855	0.064
Strict MI	937.15	160	81.69	32	< 0.001	0.069	0.845	0.010

Configural MI: same factor structure across groups; Metric MI: configural MI + equal factor loadings across groups; Scalar MI: metric MI + equal item intercepts across groups; Strict MI: scalar MI + equal residual variances across groups; best fitting MI models based on RMSEA < 0.08, CFI > 0.90, and ΔCFI < 0.01 compared to lower-level MI are shown in bold.

Intercorrelated RWA and SDO mean scores (*r* = .42), uncorrelated RWA and SDO factor scores based on the Anderson-Rubin factor-score estimation method (DiStefano et al. 2009), and latent RWA and SDO variables corrected for item specificity including random error of measurement were used in the ongoing twin family analyses. The raw means and variances of these scores for different zygosity groups, birth cohorts of twins, and family members are shown in the supplementary Table S1 (see https://osf.io/mq83d/). Generally, the adolescent siblings tended to show higher RWA and SDO scores compared to older birth cohorts, while the variance in RWA and SDO tended to increase across cohorts.

### Analyses

The preparative psychometric analyses subsumed in the previous section (see the corresponding HTML-file ‘Psychometric Analyses’ at https://osf.io/mq83d/ for the entire output) and the main analyses were run with the statistical software package JASP, version 0.18.3 (JASP Team 2024). JASP is a user-friendly and open-source program whose analyses are based on R code. Hence, structural equation modeling was based on lavaan syntax and NTFMs as well as MC-NTFMs were fitted to the data on the basis of full information maximum likelihood estimation to handle missing data (Bentler 2006; Little and Rubin 2019).

#### Family Correlation Analyses

First, we approached the family similarity by calculating correlations of mean scores, factor scores, and latent variable scores of RWA and SDO, respectively, for each set of family member dyads based on the full sample and based on the families from the three different twin birth cohorts separately. The inspection of family similarity provides an initial insight into the roles of genetic and environmental sources of differences in RWA and SDO.

#### Nuclear Twin Family Model Analyses

**Fig. 1:**
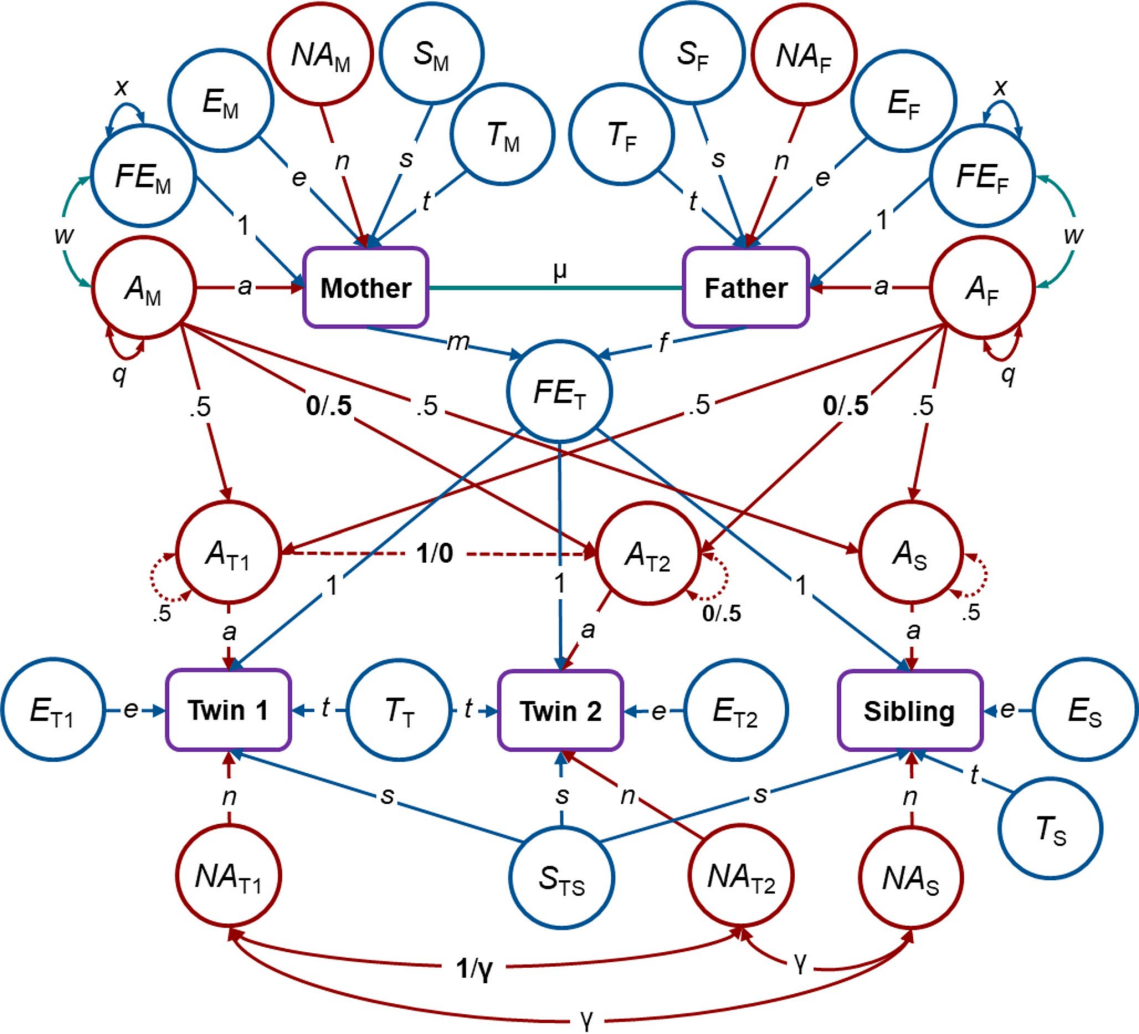
Nuclear Twin Family Model: Violet rectangles reflect the observed variance in either RWA or SDO for each set of family members; red circles refer to genetic factors; blue circles refer to environmental factors; green paths refer to specific covariances between twins’ parents and between genetic and environmental factors; bold parameters indicate that the estimates are set to be different for monozygotic twins (before the slash) than for dizygotic twins (after the slash); *A* (*a*), additive genetic factors (effects); *NA* (*n*), nonadditive genetic factors (effects) due to allelic dominance (γ = 0.25) or gene-by-gene interaction (γ = 0); *S* (*s*), sibling-specific environmental factors (effects); *T* (*t*), twin-specific environmental factors (effects); *FE*, environmental factors due to the family environment provided by parents; *E* (*e*), individual-specific environmental factors (effects); *w*, covariance between *A* and *FE* that can be interpreted in terms of passive genotype-environment covariance; µ, assortative mating; *m*, maternal non-genetic transmission; *f*, paternal non-genetic transmission; *q*, variance of *A*; *x*, variance of *FE*; variances of all other latent variables are fixed to 1

To estimate the contributions of genetic factors, environmental factors and passive *r*GE to individual differences in RWA and SDO, NTFM analyses were applied (Heath et al. 1985; Keller et al. 2009a, b). Here, we utilized data from MZ and DZ twins reared together, non-twin full-siblings, and their biological parents to decompose the observed variance in the respective constructs into components due to genetic and environmental sources (see Fig. 1).

Genetic sources can be decomposed into additive genetic factors (*A*) and nonadditive genetic factors (*NA*), with the latter either arising from allelic dominance within gene loci or gene-gene interactions, also called emergenesis or epistasis (Lykken 2006). All genetic factors are perfectly correlated between MZ twins, whereas *A* factors’ correlation is 0.5 for other first-degree relatives and *NA* factors are not correlated among other relatives, except the correlation of nonadditive genetic factors due to allelic dominance, which is 0.25 for non-MZ first-degree siblings.

Environmental sources can be broken down into influences shared by twins only (*T*), influences shared by all siblings (*S*), family environmental influences (*FE*) transmitted from parents to their offspring, and individual-specific environmental influences (*E*) not shared by family members, with the latter including random error of measurement, if not controlled for. In addition, the covariance between *A* and *FE* (*w*) can be regarded as an estimate that represents passive *r*GE, as *w* captures the extent to which parental phenotypes are genetically linked to the offspring’s phenotype and associated with the environment provided by the parents (Keller et al. 2009a, b). All these different sources of variance were estimated while considering the effects of assortative mating (µ). This was done because, as noted, assortative mating can increase genetic similarity between parents and their children as well as among their children, except for already genetically identical MZ twins, and can thus affect genetic variance (Heath and Eaves 1985).

The NTFM does not have sufficient information to estimate, in the presence of each other, nonadditive genetic factors, environmental factors shared by all offspring due to parental influences, sibling-specific common environmental influences not shared with parents, and twin-specific shared environmental influences (Keller and Coventry 2005). For that reason, we tested NTFMs assuming either nonadditive genetic effects or sibling-specific environmental influences to be zero, further specifying nonadditive genetic effects as being either due to allelic dominance or due to epistatic gene-gene interaction (epistasis). We compared the three models descriptively to one another using the Akaike Information Criterion (AIC; Akaike 1987) and the Bayesian Information Criterion (BIC; Schwarz 1978) to decide which of them fitted the data best. The respective best fitting model was used to derive the single parameter estimates, whose statistical significance was evaluated. See Hufer et al. (2020) for more details on the NTFMs used in this study and Instinske and Kandler (2024) for NTFM user tutorials based on JASP.

Based on the model parameter estimates of the best fitting model, we calculated the variance components of RWA and SDO measures as follows: The phenotypic variance can be decomposed into $a^{2} q + n^{2} + x + t^{2} + s^{2} + e^{2} + 2aw$, where ${\text{a}}^{2} q$ reflects the genetic component due to additive genetic effects, ${\text{n}}^{2}$ represents the genetic component due to nonadditive genetic effects, $x\, = \,m^{2} \, + \,f^{2} \, + \,2mf\mu$ (if phenotypic variance is standardized, i.e. $\:VAR=1$) is the environmental variance attributable to environmental transmission from parents to their offspring, ${\text{t}}^{2}$ reflects the environmental component due to twin-specific influences, ${\text{s}}^{2}$ represents the environmental component attributable to sibling-specific influences, ${\text{e}}^{2}$ is the individual-specific environmental component, and 2*aw* is the component due to passive *r*GE (Hufer et al. 2020; Keller et al. 2009a, b).

#### Multi-Cohort Nuclear Twin Family Model Analyses

After investigating the contributions to the variance in RWA and SDO scores for the entire twin family sample, we scrutinized the role of *r*GE for the three age cohorts of twins. We specified a MC-NTFM using the three age cohorts as different groups. For an initial assessment of whether the contribution of passive *r*GE to individual differences decreased between adolescence and adulthood (Hypothesis 1) and the relevance of active or evocative *r*GE increased (Hypothesis 2), we compared MC-NTFMs including different constraints across the three age cohorts to another using the likelihood ratio test (Δχ²): First, we examined if a MC-NTFM in which the genetic variance components (${\text{a}}^{2} q$ and ${\text{n}}^{2}$) as well as the passive *r*GE ($\:2aw$) component were constrained to be equal across cohorts fitted the data significantly worse than a corresponding MC-NTFM in which all parameters were estimated freely for different cohorts. Second, we examined if a MC-NTFM in which all parameters were constrained to be equal fitted the data significantly worse than the two less constrained models. If so, we ascertained which of the model parameters were responsible for potential differences between cohorts. See also https://osf.io/mq83d/ for lavaan codes of the NTFMs and MC-NTFMs.

## Results

### Family Correlations

We estimated the correlations of different family member dyads for correlated mean scores, orthogonal factor scores, and latent variable scores of RWA and SDO. This was done for the full sample and the three separate family groups with different twin birth cohorts. All detailed family correlations including 95% confidence intervals can be found at https://osf.io/mq83d/ in the HTML-file ‘Family Correlations’.

**Fig. 2:**
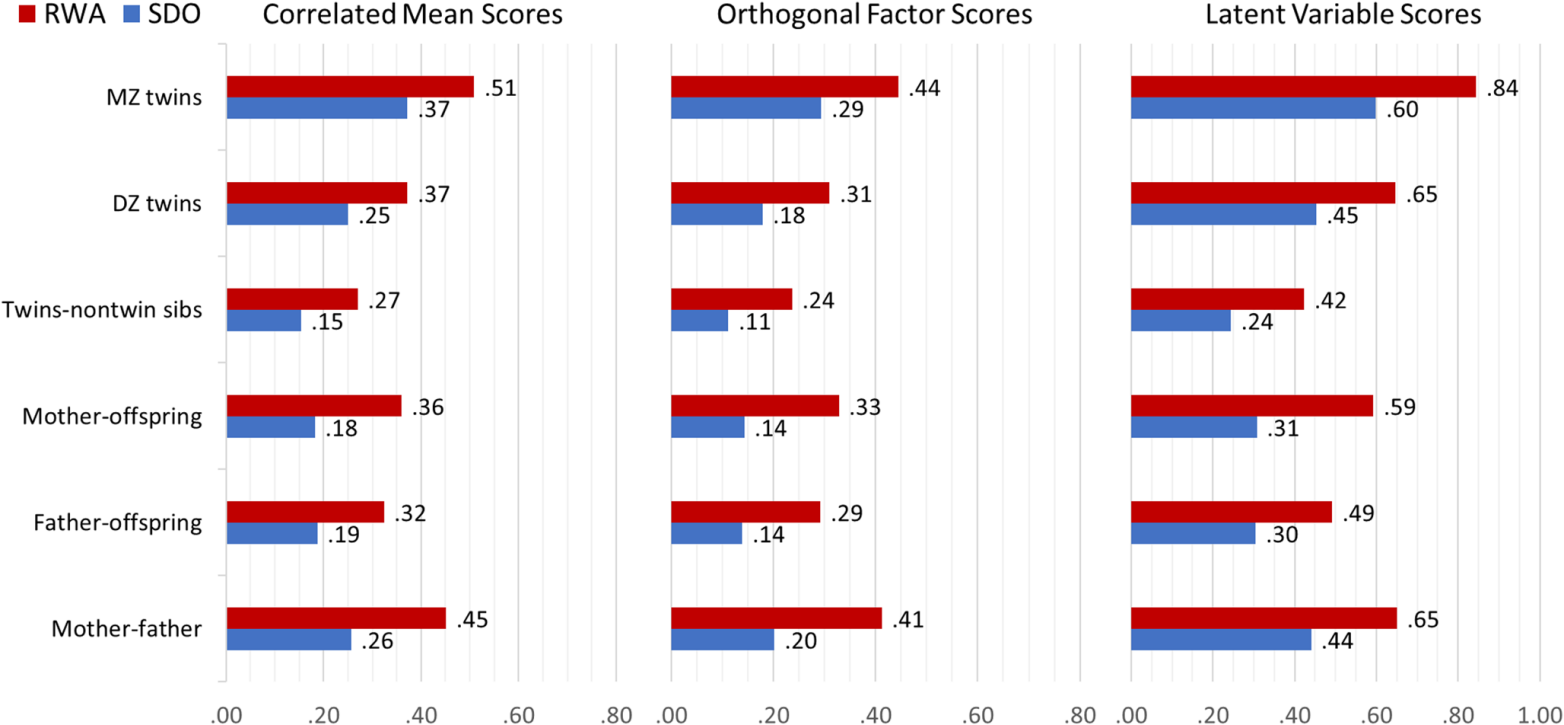
Family correlations for correlated RWA and SDO mean scores (*r* = .42), uncorrelated factor scores, and latent variable scores based on structural equation modeling; *MZ* Monozygotic, *DZ* Dizygotic

Figure 2 summarizes the family correlations for the family dyads relevant in the nuclear twin family design. The parent-offspring and the twin-nontwin sibling correlations are weighted averages across all potential combinations (i.e., across parent-twin a, parent-twin b, and parent-nontwin sib, as well as across twin a-nontwin sib and twin b-nontwin sib). The orthogonal factor scores yielded lower family correlations, which is not surprising, given that the common variance in RWA and SDO has been shown to be primarily genetic in nature (Nacke and Riemann 2023). Adjusting RWA and SDO factors for this common component would result in reduced family correlations compared to those based on correlated mean scores (−[0.07-0.08] for MZ twins and −[0.03-0.06] for other core family relatives). Latent variable scores provided the highest family correlations, because they are not attenuated due to item-specific method variance and random measurement error variance.

The general patterns of family correlations were comparable across all three kinds of measures. Family resemblance and spouse similarity was higher for RWA than SDO, indicating larger influences shared by family members on variance in RWA and larger individual-specific environmental effects on variance in SDO. In all cases, the MZ twin correlations were higher than the DZ twin correlations, indicating contributions of genetic factors to individual differences in both RWA and SDO. The DZ twin correlations tended to be higher compared to the twin-nontwin sibling and the parent-offspring correlations, suggesting environmental influences that are shared by twins only. Mother-father correlations were moderate to substantial, indicating assortative mating, which seems to play a larger role for RWA compared to SDO given the higher mother-father correlation.

**Fig. 3:**
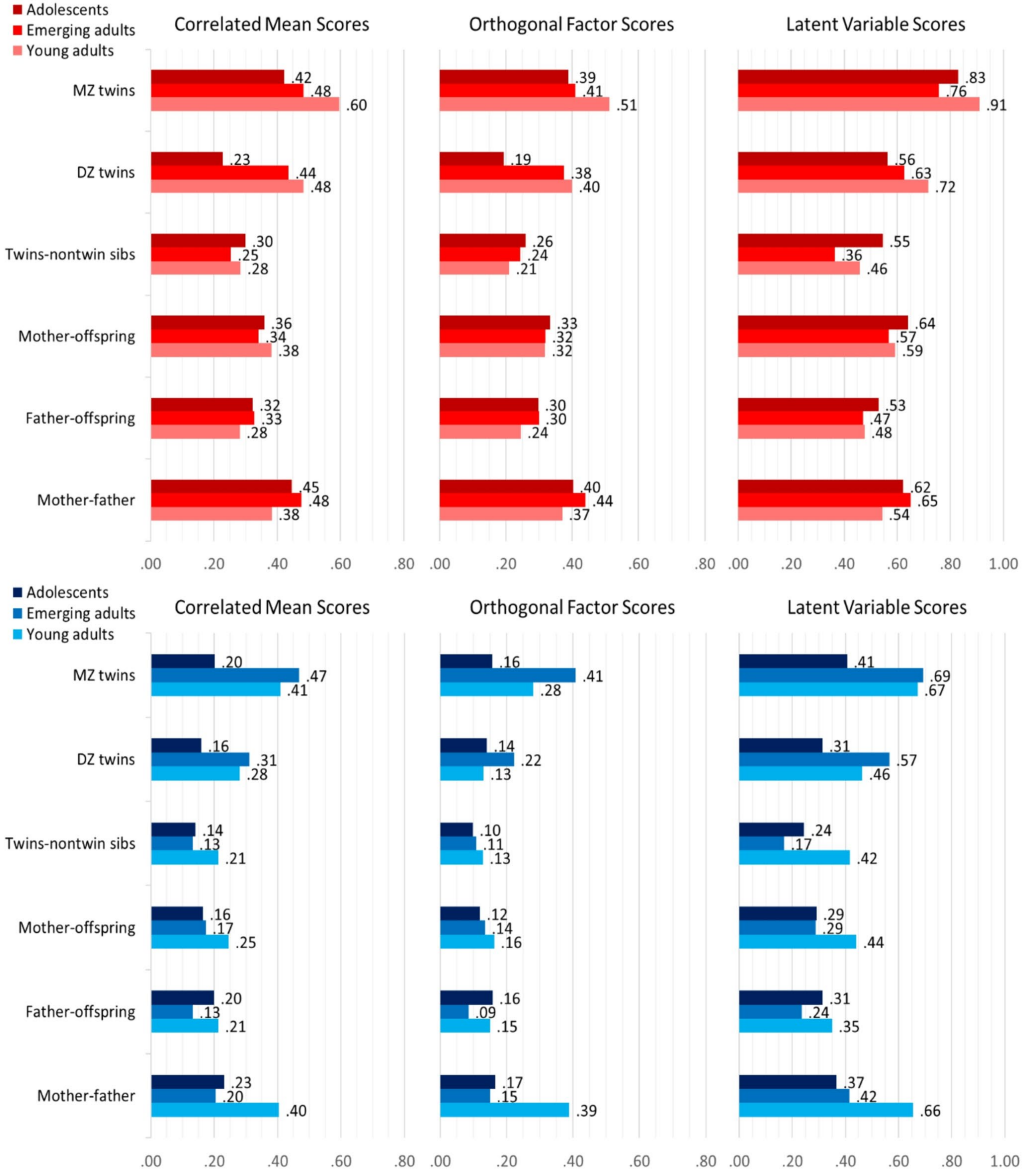
Cohort-specific family correlations for correlated RWA (top/red) and SDO (bottom/blue) mean scores, uncorrelated factor scores, and latent variable scores based on structural equation modeling; *MZ* Monozygotic, *DZ* Dizygotic

An inspection of the cohort-specific family correlations (see Fig. 3) yielded the highest twin correlations in RWA for the oldest cohort of young adult twins compared to emerging young adult (21-year old) and adolescent (15-year old) twins. For SDO, the highest twin correlations were consistently found for the emerging adult twins compared to the other cohorts. These differences indicate lower twin resemblance in RWA and SDO for the adolescent twin cohort compared to adult twins. Other family correlations did not vary that systematically, except the spouse correlations in SDO, which were substantially higher in families with the oldest twin cohorts. The latter is probably a sample-specific deviation. The NTFMs can deal with those deviations which otherwise would lead to more or less biased estimates of genetic and environmental contributions to the variance in RWA and SDO.

### Nuclear Twin Family Modeling

**Fig. 4:**
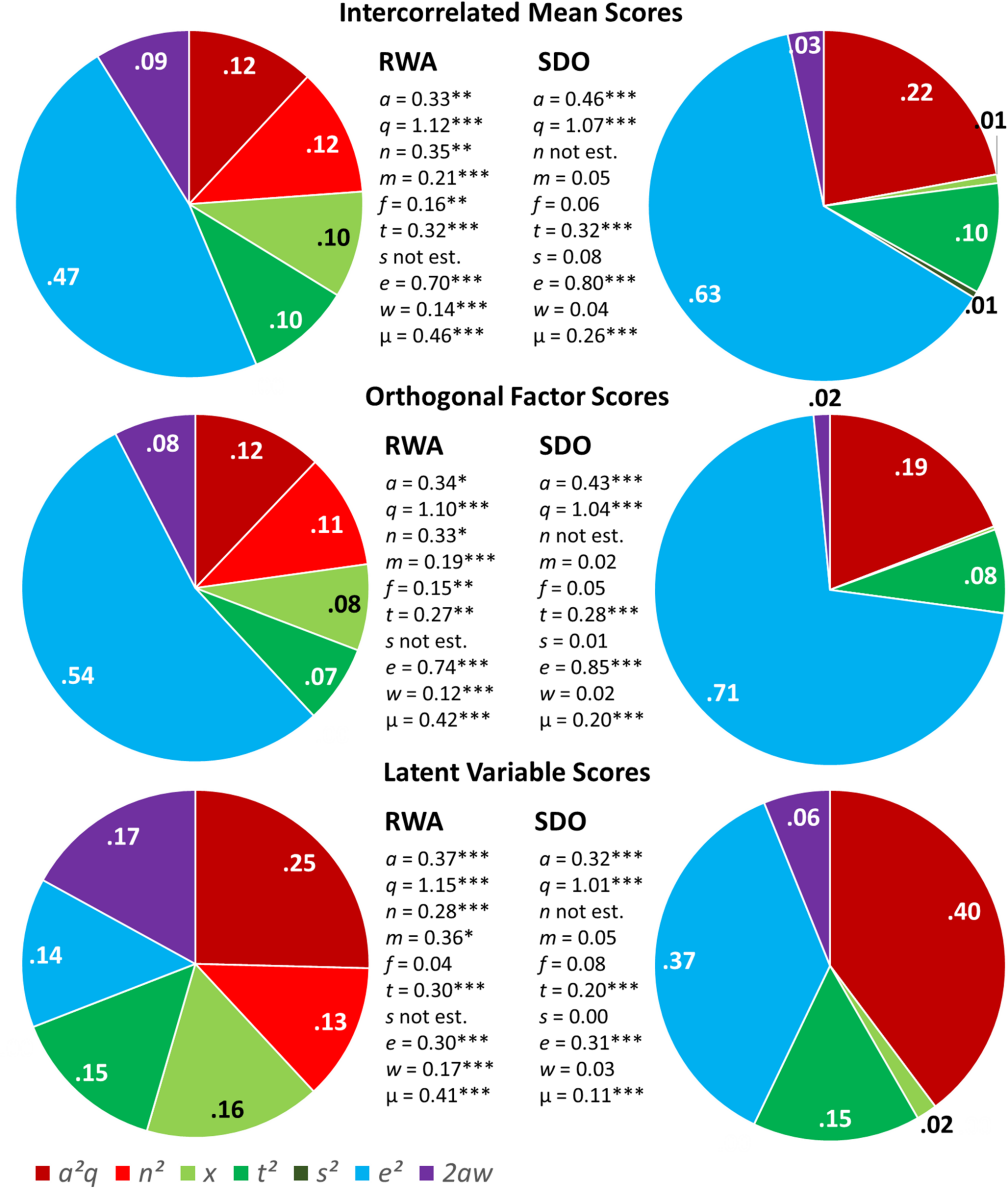
Nuclear Twin Family Model results (see also Fig. 1): Variance components that add up to 1 are shown in the pie charts; $\:a$, additive genetic effect; $\:q$, variance of additive genetic factors; $\:n$, nonadditive genetic effect; $\:m$, maternal non-genetic transmission effect; $\:f$, paternal non-genetic transmission effect; $\:t$, twin-specific shared environmental effect; $\:s$, sibling-specific shared environmental effect; $\:e$, individual-specific environmental effect; $\:w$, passive genotype-environment covariance; $\:{\upmu\:}$, spouse covariance; ${\text{a}}^{2} q$, additive genetic component; ${\text{n}}^{2}$, nonadditive genetic component; $\:x$, variance of environmental factors due to the family environment provided by parents; ${\text{t}}^{2}$, variance due to twin-specific shared environmental effects; ${\text{s}}^{2}$, variance due to shared environmental effects common to all siblings; ${\text{e}}^{2}$, variance due to individual-specific environmental effects; $\:2aw$, variance due to passive genotype-environment correlation; ***$\:p<.001$; **$\:p<.01$; *$\:p<.05$; see NTFMs_RWA_SDO.html output file and Table S2 at https://osf.io/mq83d/ for exact $\:p$-values and confidence intervals

Descriptive model comparisons of different NTFMs either allowing for sibling-specific shared environmental influences or nonadditive genetic effects due to epistasis or allelic dominance yielded fairly consistent results across the different measures of RWA and SDO. The NTFM allowing for nonadditive genetic effects instead of sibling-specific shared environmental influences on RWA variance showed descriptively the best model fit, whereas all three models provided almost identical fit statistics and indices for SDO (see Table 3; additional fit measures and all model parameter estimates including exact *p*-values and confidence intervals can be found in the NTFMs_RWA_SDO.html file and Table S2 at https://osf.io/mq83d/). Based on the model parameter estimates of the best fitting model, we calculated the variance components of RWA and SDO measuresThese variance components are summarized in Fig. 4.

**Table 3:** Nuclear twin family model (NTFM) fit statistics and indices based on different RWA and SDO measures

NTFMs and measures	Fit statistics/indices						
	AIC	BIC	χ²	*df*	*p*	CFI	RMSEA
RWA z-standardized mean score							
Sibling effects	13638.12	13680.30	36.94	32	0.251	0.994	0.015
Epistasis	13637.37	13679.55	36.19	32	0.279	0.995	0.013
Allelic dominance	13636.51	13678.69	35.33	32	0.314	0.996	0.012
RWA orthogonal factor score							
Sibling effects	13791.90	13834.08	34.07	32	0.368	0.997	0.009
Epistasis	13791.44	13833.62	33.62	32	0.389	0.998	0.008
Allelic dominance	13790.95	13833.13	33.12	32	0.412	0.998	0.007
RWA latent variable score							
Sibling effects	59126.00	59605.78	778.48	369	< 0.001	0.876	0.039
Epistasis	59123.73	59603.52	776.21	369	< 0.001	0.877	0.039
Allelic dominance	59126.00	59605.78	778.48	369	< 0.001	0.876	0.039
SDO z-standardized mean score							
Sibling effects	14156.60	14198.77	32.50	32	0.442	0.988	0.005
Epistasis	14156.61	14198.79	32.51	32	0.442	0.988	0.005
Allelic dominance	14156.61	14198.79	32.51	32	0.442	0.988	0.005
SDO orthogonal factor score							
Sibling effects	14273.13	14315.31	28.77	32	0.631	1.000	0.000
Epistasis	14273.13	14315.31	28.77	32	0.631	1.000	0.000
Allelic dominance	14273.13	14315.31	28.77	32	0.631	1.000	0.000
SDO latent variable score							
Sibling effects	59856.08	60335.87	668.74	369	< 0.001	0.835	0.034
Epistasis	59856.08	60335.87	668.74	369	< 0.001	0.835	0.034
Allelic dominance	59856.08	60335.87	668.74	369	< 0.001	0.835	0.034

NTFM with sibling effects: $\:s\ne\:0$ and $\:n=0$; NTFM with epistasis: $\:n\ne\:0$ (monozygotic twin correlation: 1, dizygotic twin/nontwin sibling correlation: 0) and $\:s=0$; NTFM with allelic dominance: $\:n\ne\:0$ (monozygotic twin correlation: 1, dizygotic twin/nontwin sibling correlation: 0.25) and $\:s=0$; AIC: Akaike Information Criterion; BIC: Bayesian Information Criterion; *df*: degrees of freedom; CFI: Comparative Fit Index; RMSEA: Root Mean Square Error of Approximation

The analyses yielded consistent differences across the three measures regarding the sources of variance in RWA and SDO. While passive *r*GE, environmental parental transmission, and nonadditive genetic effects played a significant role for variance in RWA, none of these factors significantly contributed to the variance in SDO. Even after correction for item-specific and random error variance in the latent variable models, individual-specific environmental influences contributed substantially to the variance in SDO (37%), as compared to a small but significant contribution to individual differences in RWA (14%). There were also some similarities across the two constructs: The entire (i.e., additive and nonadditive) genetic variance and the variance attributable to twin-specific shared environmental influences were comparable in size for RWA and SDO.

### Multi-Cohort Nuclear Twin Family Modeling

In a final step, we tested for differences between twins’ birth cohorts regarding genetic and environmental variance components of RWA and SDO. We found that the NTFM, which takes into account nonadditive genetic effects in the presence of additive genetic effects, led to wide confidence intervals for estimates of corresponding model parameters $\:a$ and $\:n$ (particularly in case of allelic dominance, see Table S2). This indicates low statistical power for the estimation of both parameters in the presence of each other. Therefore, we decided to use a reduced MC-NTFM, in which sibling-specific shared environmental influences and nonadditive genetic factors were assumed to be negligible, for both RWA and SDO. We considered this constraint to be appropriate because the NTFMs implied that sibling-specific shared environmental effects were negligible for RWA and SDO. Furthermore, it is generally assumed that the estimates of additive genetic contributions approximately cover the sum of additive and non-additive contributions, even if the latter are not estimated for complex traits (Hill et al. 2008).

**Fig. 5:**
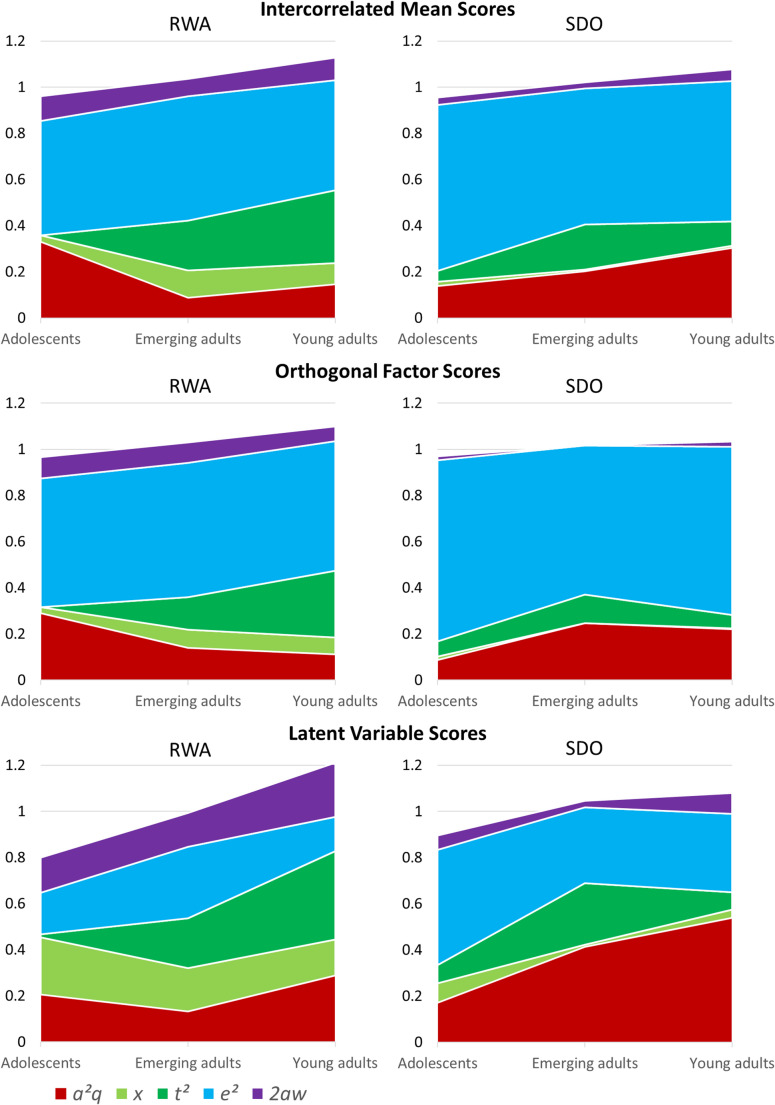
Multi-Cohort Nuclear Twin Family Model results: Variance components for different twin cohorts are shown in the stacked surface plots; ${\text{a}}^{2} q$, genetic component; $\:x$, variance of environmental factors due to the family environment provided by parents; ${\text{t}}^{2}$, variance due to twin-specific shared environmental effects; ${\text{e}}^{2}$, variance due to individual-specific environmental effects; $\:2aw$, variance due to passive genotype-environment correlation. The phenotypic variances were standardized across cohorts. See the MC-NTFMs HTML-output files and Table S3 at https://osf.io/mq83d/ for exact $\:p$-values and confidence intervals

The cohort differences in standardized variance components are summarized in Fig. 5. Model comparisons revealed no significant differences in genetic components and in passive *r*GE between cohorts for all measures of RWA and SDO, based on the likelihood ratio test (see Table 4, 2:equal hypo vs. 1:different). The model test results were not in line with our hypotheses. For SDO measures, all model parameters could be constrained to be equal across cohorts without a significant decline in model fit (see Table 4, 3:equal all vs. 1:different). By contrast, cohort differences in specific variance components proved to be statistically significant for all measures of RWA, especially after genetic and *r*GE components had been restricted to equal values (see Tables 4, 3:equal all vs. 2:equal hypo).

When screening specific model parameters (see supplementary Table S3 at https://osf.io/mq83d/), it turned out that twin-specific shared environmental influences drive the cohort differences in RWA: While not important for adolescent twins, they were important (statistically significant) for adult twins. As can be clearly seen in Fig. 5, this component increased across all cohorts and thus appears to be responsible for the increasing variance in RWA. Restricting this variance component to be equal across cohorts led to a significant decline in model fit for all measures of RWA (Δχ² ≥ 8.47, Δ*df* = 2, *p* ≤ .015; see MC-NTFM output files with appendix “equalT” at https://osf.io/mq83d/). Although Fig. 5 also suggests that other components tended to vary between cohorts, these differences were not statistically significant.

**Table 4:** Multi-Cohort nuclear twin family model (MC-NTFM) fit statistics and model comparisons based on different RWA and SDO measures

MC-NTFMs and measures	Fit statistics/indices							
	AIC	χ²	*df*	*p*	Test	Δχ²	Δ*df*	*p*
RWA z-standardized mean score								
1:different	13616.11	127.41	99	0.029				
2:equal hypo	13612.43	131.73	103	0.030	2 vs.1	4.32	4	0.365
3:equal all	13622.89	158.19	111	0.002	3 vs.1	30.78	12	0.002
					3 vs.2	26.46	8	< 0.001
RWA orthogonal factor score								
1:different	13783.63	113.97	99	0.144				
2:equal hypo	13778.09	116.44	103	0.172	2 vs.1	2.47	4	0.651
3:equal all	13779.94	134.29	111	0.066	3 vs.1	20.32	12	0.061
					3 vs.2	17.85	8	0.022
RWA latent variable score								
1:different	59113.97	1967.05	1116	< 0.001				
2:equal hypo	59109.81	1970.89	1120	< 0.001	2 vs.1	3.84	4	0.428
3:equal all	59127.33	2004.41	1128	< 0.001	3 vs.1	37.37	12	< 0.001
					3 vs.2	33.53	8	< 0.001
SDO z-standardized mean score								
1:different	14149.43	124.87	99	0.040				
2:equal hypo	14143.81	127.25	103	0.053	2 vs.1	2.37	4	0.667
3:equal all	14144.68	144.12	111	0.019	3 vs.1	19.25	12	0.083
					3 vs.2	16.87	8	0.031
SDO orthogonal factor score								
1:different	14273.69	117.75	99	0.096				
2:equal hypo	14267.29	119.34	103	0.129	2 vs.1	1.59	4	0.810
3:equal all	14265.48	133.53	111	0.072	3 vs.1	15.79	12	0.201
					3 vs.2	14.19	8	0.077
SDO latent variable score								
1:different	59814.48	1671.91	1116	< 0.001				
2:equal hypo	59810.66	1676.09	1120	< 0.001	2 vs.1	4.18	4	0.382
3:equal all	59809.36	1690.78	1128	< 0.001	3 vs.1	18.87	12	0.092
					3 vs.2	14.69	8	0.065

MC-NTFMs with 1: different model parameters ($\:a$, $\:m$, $\:f$, $\:t$, $\:e$, and $\:{\upmu\:}$) and nonlinear constraints ($\:q$, $\:w$, and $\:x$) across cohorts of twins, 2: equal variance components relevant for hypotheses (${\text{a}}^{2} q$ and 2$\:aw$) across cohorts, and 3: equal model parameters incl. nonlinear constraints across cohorts; AIC: Akaike Information Criterion; RMSEA: Root Mean Square Error of Approximation; *df*: degrees of freedom; Δχ²: likelihood ratio

## Discussion

The current study examined different genetic and environmental sources of individual differences in RWA and SDO and whether their importance varies between these two constructs and across three age cohorts. For this purpose, we used extended twin family data encompassing families with three different twin birth cohorts. Our twin family model analyses yielded new insights into both similarities and differences regarding the sources of variance in RWA compared to SDO, and how these variance components vary across different age cohorts.

### Different Sources for RWA and SDO?

Our study indicates that there were no substantive differences between RWA and SDO in terms of total genetic variance. This finding is consistent with some previous studies (e.g., Kleppestø et al. 2024), but contradicts other findings based on a different German twin family sample that included older twins which suggested a smaller genetic component for SDO compared to RWA (Kandler 2015) or even a negligible genetic component after exclusion of the common variance with RWA (Kandler et al. 2016). However, differences regarding genetic influences could be observed between RWA and SDO when considering the additive and nonadditive genetic variance components separately. Both additive and nonadditive genetic sources of variance were statistically significant for RWA, while only additive genetic sources were relevant for individual differences in SDO.

Compared to models based on data from twins only, employing an NTFM provides more precise estimates of the genetic components when there are also relevant environmental influences shared by twins (Instinske and Kandler 2024). In our study, we found that after taking into account assortative mating, which was present for both RWA and SDO but tended to be higher for RWA in line with previous studies (e.g., Kandler et al. 2015), environmental transmission from generation to generation was statistically significant for RWA, but not for SDO. This meant that passive *r*GE was only relevant for RWA, with no statistically significant differences between the age cohorts. This finding is consistent with a previous German twin family study using data from an older sample of twins and their family members (Kandler et al. 2016). About 16% of the variance in RWA in the previous study and 17% in the current study could be attributed to passive *r*GE after correcting the total variance for measurement error variance. Generally, RWA exhibited a higher familial resemblance, which can be attributed to larger environmental influences shared among family members. In contrast, SDO appears to be substantially more influenced by individual-specific environmental effects, even after adjustments for measurement error variance. Accordingly, individual differences in RWA may be more malleable by within-familial sources, while extra-familial circumstances may represent the primary environmental determinants of variance in SDO.

Our findings also indicate that twin-specific shared environmental factors play a crucial role in shaping both RWA and SDO. This suggests a potential impact of age-related effects among twin siblings or, more plausibly, a twin-specific co-development process in which same-age siblings influence each other’s socio-political preferences and attitudes more strongly than siblings of different ages. Consistent with the latter interpretation is the observation that components due to twin-specific shared environmental effects tended to be lower (but not negligible) when based on the orthogonal factor scores compared to the mean or latent variable scores of both RWA and SDO. This indicates that some proportions of twin-specific shared environmental effects might account for a considerable amount of the common variance in RWA and SDO, and thus yield a common impact on both constructs.

How might all of these findings, taken together, be explained? We propose that at both the societal level and the level of families and individuals, RWA is rooted in processes of *within-group cohesion*, whereas SDO is more closely tied to *between-group competition and hierarchy*. The RWA items used in this study emphasize law and order, obedience, and the suppression of dissent, which are orientations that can, under certain circumstances, foster social cohesion. Similar patterns may appear in families and other small groups characterized by traditional or fundamentalist religious values, which are strongly correlated with RWA (Womick et al. 2022). It is also noteworthy that these values emphasize conformity, discipline, and moral regulation, which have an affinity to RWA. Because family members are highly interdependent, disagreements over these cohesion-related outlooks are likely to produce conflict. For this reason, individuals may prefer or remain with long-term partners who share their perspectives on these matters, creating a basis for assortative mating, which as noted was higher for RWA than SDO.

It is possible, however, that assortative mating occurs less on the basis of RWA itself than on the shared religious commitments and broader traditionalist worldviews that are consistent with and may even conceptually overlap with RWA. Indeed, there is evidence for robust assortative mating on religion and worldview (Kalmijn 1998; Myers 2006), which can stabilize marriages by reinforcing shared value commitments. By contrast, SDO-related orientations, such as preferences for group dominance and group hierarchies, are less central to spousal relations and family functioning. Instead, they are more relevant in broader social contexts characterized by intergroup competition, making them less salient to within-family dynamics and mate selection.

This framework may also explain why passive rGE and environmental parent transmission appear to be stronger for RWA than for SDO. Parents not only transmit genetic predispositions toward authoritarianism but may also construct family environments through religious practices and related traditions that align with RWA. In contrast, although parents may pass along genetic dispositions toward group dominance or group competition, these orientations are typically expressed in extrafamilial contexts. This could explain why SDO shows weaker shared environmental effects and stronger individual-specific influences. This distinction may also help to account for the higher overall family resemblance observed for RWA compared with SDO.

### Different Sources for Different Ages?

Generally, our study implied that individual differences in RWA and SDO tend to increase as individuals age. In the light of developmental theories (Scarr 1992; Scarr and McCartney 1983), these trends could reflect the increasing importance of individual experiences and environments as well as the individualizing interdependence between genetic and environmental factors: As people grow older and mature, such factors may play a more decisive role in shaping their socio-political attitudes. Initially, we hypothesized that passive *r*GE declines (Hypothesis 1), while genetic differences increase with age potentially reflecting active *r*GE (Hypothesis 2) for both RWA and SDO. Our study found that these preregistered hypotheses could not be confirmed. The results revealed that passive *r*GE components were generally negligible for SDO. Furthermore, overall levels of genetic variance and passive *r*GE components remained fairly constant for RWA across cohorts. Although levels of genetic differences generally tended to increase for SDO, these differences were not statistically significant.

Interestingly, we found a statistically significant increase in the twin-specific shared environmental component for RWA across the three cohorts. And for SDO, an increasing trend was observed from adolescence to emerging adulthood. For both SDO and RWA, twin-specific shared environmental influences were negligible for adolescent twins, but statistically significant for emerging adults (see Table S3), who are eligible to vote from the age of 18 and thus have a say in political life. Although not expected, these findings seem to be consistent with the idea that, as they transition from adolescence to adulthood, same-age twin siblings may talk more on political, particularly RWA-related, issues with each other and with their peers. Since interactions with peers are thought to play an important role in the shaping of political attitudes (e.g., Dey 1997), the increase in the twin-specific shared environmental component could be reflective of an increased political engagement with peers (and the twin sibling as a special peer) with increasing age. Peer or co-twin influences, which act to increase twin similarity compared to other family members, may reflect the presence of important social role models, which could have a significant impact on socio-political attitudes during the formative years of political development. These interpretations align with the conclusions drawn by Kandler et al. (2012) that environmental influences within but not across generations provide relevant sources of political orientations. Given the apparently important role of twin-specific shared environmental influences, understanding the specific underlying processes could, prospectively, provide insights into the development and evolution of socio-political attitudes.

As an alternative interpretation, twin-specific shared environmental effects could potentially mimic maturity-related genetic differences affecting both RWA and SDO. However, the fact that non-twin siblings of a twin pair were on average of a comparable age and that sibling-specific influences were negligible contradicts this interpretation. Generally, the influences of within-familial environmental factors on RWA appear to increase with age. Conversely, these influences are small for SDO and tend to decline in young adults. These findings again emphasize the differing dynamics of environmental influences on the development of the two socio-political characteristics RWA and SDO over time.

### Limitations and Future Directions

Despite the advantages of our study, including the application of a twin family design to disentangle various genetic and environmental variance components, the use of data from different age cohorts from adolescence to adulthood, a substantial sample size, and the differentiation between mean, orthogonal factor, and latent variable scores of RWA and SDO, some limitations also exist. One limitation of our study is the statistical power for detecting cohort differences. Although NTFMs allow for the estimation of multiple potential influences on variance beyond the classical twin model, and Fig. 5 suggests age differences in several genetic and environmental components (e.g., an increase in genetic differences in SDO with age), the model comparison tests did not reveal any statistically significant cohort differences, with the exception of the twin-specific shared environmental components of RWA. Future multi-cohort twin family studies with larger sample sizes per cohort may detect more differences.

A further limitation is the inability to disentangle between cohort and age effects. In other words, we could not clearly differentiate whether differences between the three different age groups were due to genuinely chronological age-related changes (age effects), or if they were related to the specific time period in which the different cohorts were born and raised (cohort effects). This limits the implications of our study for an understanding of the development of RWA and SDO. To address this limitation, subsequent studies could utilize data from multiple timepoints in addition to data from multiple birth cohorts to apply longitudinal twin family model analyses.

Another limitation in our study was the lack of consideration for sex effects. Sex, as a biological variable, can significantly influence the manifestation of both RWA and SDO due to variations in hormone levels, societal influences, and other sex-specific factors. Future studies should therefore seek to incorporate opposite-sex DZ twins for sex limitation modeling (Neale et al. 2006) which might provide a more exhaustive and nuanced understanding of the genetic and environmental influences on RWA and SDO.

The different ages of family members in an NTFM also represents a limitation in our study. NTFMs require to assume that the genetic and environmental variance is the same across the different generations. This might lead to distortions, as such an assumption may not be tenable, especially since socio-political preferences and attitudes may be influenced by different social contexts experienced by different generations (Zubielevitch et al. 2023). Although data collection of family members when they are in the same age or developmental phase may be difficult to realize, this would enable a more comprehensive understanding of the transmission of RWA and SDO from one generation to the next. At least in the case of non-twin siblings, we would expect lower distortions due to the fact that they are on average at developmental stages comparable to those of the twins.

Finally, our measures of RWA and SDO were rather short ones, as is typically the problem in large panel studies. The factor loadings were in part rather low (with standardized estimates ranging between 0.25 and 0.69), indicating substantive item specificity for some items. Although factorial validity and metric measurement invariance could be established for the measures used, future studies should use multi-item and multi-faceted measures of RWA and SDO to further improve internal reliability and to examine these constructs at the facet level.

## Conclusion

The manifestations of both RWA and SDO appear to be to some extent similar within families, although spouse similarity and family resemblance across all core family members appear to be consistently higher for RWA than for SDO. Our nuclear twin family model analyses suggest that the higher family resemblance in RWA is attributable to significant environmental transmission from parents to offspring and passive genotype-environment correlation. For SDO, however, these two influences turned out to be negligible, such that individual-specific environmental circumstances seemed to primarily account for differences in SDO. Although additive and nonadditive genetic sources appear to drive individual differences in RWA and only additive genetic sources seem to have an impact on individual differences in SDO, the total genetic variance did not differ between RWA and SDO.

Individual differences and twin resemblance in RWA and SDO tended to be smaller for adolescents compared to emerging adult and young adult twins. However, contray to our expectation, especially for RWA, the larger variance in adulthood was not attributable to larger genetic differences as a consequence of an increasing importance of active and evocative genotype-environment correlation. Rather, the larger variance with age was the result of an increasing importance of twin-specific shared environments. These findings point to the relevance of the social environment common to twins but not to other siblings during the formative years of socio-political development that mainly takes place as adolescents mature into adulthood. These environmental influences shared by twins could include having the same friends or mentors, or even the influences that same-age twins have on each other.

## Data Availability

The raw means and variances of these scores for different zygosity groups, birth cohorts of twins, and family members are shown in the supplementary Table S1 (see https://osf.io/mq83d/).
